# The evidence for the effectiveness of safety alerts in electronic patient medication record systems at the point of pharmacy order entry: a systematic review

**DOI:** 10.1186/1472-6947-13-69

**Published:** 2013-07-01

**Authors:** Oluwagbemileke Ojeleye, Anthony Avery, Vaibhav Gupta, Matthew Boyd

**Affiliations:** 1 Division of Social Research in Medicines and Health, University of Nottingham, Nottingham, UK; 2 Division of Primary Care, University of Nottingham, Nottingham, UK

**Keywords:** Electronic patient medication record system, Safety feature, Safety alert, Safety warning, Pharmacy order entry system, Decision support, Pharmacy computer system, Medicine supply, Drug alert

## Abstract

**Background:**

Electronic Patient Medication Record (ePMR) systems have important safety features embedded to alert users about potential clinical hazards and errors. To date, there is no synthesis of evidence about the effectiveness of these safety features and alerts at the point of pharmacy order entry. This review aims to systematically explore the literature and synthesise published evidence about the effectiveness of safety features and alerts in ePMR systems at the point of pharmacy order entry, in primary and secondary care.

**Methods:**

We searched MEDLINE, EMBASE, Inspec, International Pharmaceutical Abstracts, PsycINFO, CINHAL (earliest entry to March 2012) and reference lists of articles. Two reviewers examined the titles and abstracts, and used a hierarchical template to identify comparative design studies evaluating the effectiveness of safety features and alerts at the point of pharmacy order entry. The two reviewers independently assessed the quality of the included studies using Cochrane Collaboration’s risk of bias tool.

**Results:**

Three randomised trials and two before-after studies met our criteria. Four studies involved integrated care facilities and one was hospital-based. The studies were all from the United States (US). The five studies demonstrated statistically significant reduction in medication errors in patients with renal insufficiency, pregnant women dispensed US Food Drug and Administration (FDA) risk category D (evidence of fetal risk but therapeutic benefits can outweigh the risk) or X (evidence suggests that risk to the fetus outweighs therapeutic benefits) medication, first dispensing of inappropriate medications in patients aged 65 and above, co-dispensing of interacting drugs, and adverse drug events related to hyperkalaemia.

**Conclusions:**

This systematic review shows that the safety features of ePMR systems are effective in alerting users about potential clinical hazards and errors during pharmacy order entry. There are however, problems such as false alerts and inconsistencies in alert management. More studies are needed from other countries and pharmacy practice settings to assess the effectiveness of electronic safety features and alerts in preventing error and reducing harm to patients.

## Background

In 2011, over 950 million prescription items were dispensed in community pharmacies in England alone [1]. This number is on an upward trajectory. Coupled with the error producing and error provoking environment [2] in which pharmacists work, it means that there are numerous opportunities for medication errors to occur. It has been suggested that technologies such as electronic Patient Medication Record (ePMR) systems can help to prevent medication errors, reduce potentially inappropriate prescribing and prevent harm to patients [3,4]. However, results from systematic reviews and recent studies are indeterminate [5-8]. It has been reported that pharmacy software is less than ideal [9] and that safety alerts are often bypassed [10]. The systems have also been implicated as a cause of new risks for errors [11,12].

The medication use process in the United Kingdom (UK) comprises of four basic steps- prescribing, dispensing, administration and monitoring. In primary care, a prescription is usually generated by a general practitioner within the National Health Service (NHS) framework. The prescription is then presented at a community pharmacy contracted by the NHS for dispensing. The term patient medication record relates solely to the record of prescriptions dispensed to a patient by an individual pharmacy and covers items dispensed from NHS prescriptions, private prescriptions and very occasionally, over-the-counter medications. This record may be paper-based or kept in electronic format on electronic Patient Medication Record (ePMR) systems. These systems can be found in all primary and secondary care pharmacies in the UK. Other names used for ePMR systems in the literature include pharmacy computer system [13-15], pharmacy information system [16,17] and pharmacy information management system [18-21]. There is currently no common specification for ePMR systems in the UK.

Prescriptions issued within the NHS in the UK are usually computer generated although a prescription may also be handwritten. In primary care, it is a legal requirement for a prescription to be dispensed under the supervision of a pharmacist. For this reason, pharmacists are closely involved in the dispensing process. When a prescription is received in the pharmacy, the medication ordered by the prescriber is entered into the ePMR system. This step in the dispensing process is referred to as pharmacy order entry. The task can be performed by any qualified staff in the dispensary such as a dispenser, technician or pharmacist depending on staff availability and the workflow adopted within the respective pharmacy. A recent development in the UK is the electronic prescription service (EPS). EPS allows the electronic transmission of the prescriber’s intentions direct to a nominated dispensing pharmacy. The workflow with EPS mirrors that of physical prescriptions with the exception of manual item entry. All other steps are performed as before so the ePMR system is still required to make point of order entry checks.

In secondary care, the workflow is different. Pharmacists routinely perform clinical verifications in the ward environment prior to dispensing in the main hospital pharmacy without reference to electronic prescribing systems. Although ePMR systems have some safety features similar to those available in computerised physician order entry systems (CPOEs), the ePMR systems used in community pharmacy in the UK are not normally interfaced with other clinical information systems external to the pharmacy such as the patient’s primary care health record maintained by the general practitioner thus limiting the extent of their safety performance.

The majority of ePMR systems are stand-alone systems. Terminals may be networked together if there is more than one in a particular pharmacy. The systems are designed to support the operations of individual pharmacies. They incorporate patient-specific demographic, medication and sometimes, clinical data to support review of medication for appropriateness. They are also used for managing the inventory and can interact fully with other clinical systems if enabled.

In addition to manual safety checks conducted by pharmacists, ePMR systems are widely used in primary and secondary care to support pharmacists’ clinical decision-making and safe dispensing of medications during pharmacy order entry. The safety features embedded in them, alert users about potentially unsafe medications, drug combinations, interactions, clinical hazards, errors and adverse events. Similar technologies such as electronic prescribing systems, bar coding and automation are helping to cut down on occurrence of medication errors in other practice domains [22-24] but it has been suggested that the performance of some of these features is sub-optimal [15,25-27].

Data about the impact of safety features on pharmacists’ decision-making behaviour and medication error prevention at the point of pharmacy order entry is scant and often disparate. A number of closely related studies found in literature have focused on different perspectives such as the point of prescribing and administration [28-30]. To our knowledge, there is no published systematic review to date examining the type of safety features in ePMR systems in primary and secondary care, and their effectiveness at the point of pharmacy order entry.

To address this gap in literature, we carried out a systematic review to explore the literature and synthesise published evidence about the effectiveness of safety features and alerts in ePMR systems at the point of pharmacy order entry in primary and secondary care pharmacy settings. For this purpose, a safety feature in an ePMR system is defined as, “a feature which enables diverse pieces of clinical and patient information to be compared in order to generate patient-specific advice or alert about potential and known clinical hazards, at the pharmacy order entry stage of the medication use process thereby eliminating or minimising the risk of harm to patients”.

## Methods

### Study identification and eligibility

#### The population

Studies of patient medication record or pharmacy order entry systems used in pharmacies were included while studies about other clinical information systems and clinicians other than pharmacists were excluded.

#### The interventions

The interventions included alerts, warnings, or prompts about safety of medications, appearing on ePMR systems during pharmacy order entry or at the point of dispensing in the pharmacy. Examples are drug-drug interactions, therapeutic duplications, and allergy alerts. Drug Utilisation Review (DUR) alerts generated for non-safety reasons were excluded.

#### The outcomes

The outcomes of interest included changes in medication error, morbidity, mortality and adverse drug events. Studies with outcomes not related to safety were excluded.

#### The study types

Studies that used comparative designs to evaluate the effectiveness of ePMR systems’ safety features and alerts at the point of pharmacy order entry were included. For example, randomised controlled trials and before-after studies. Studies published only in abstract form were excluded.

#### Sources of information and search strategy

Literature searches were conducted in MEDLINE (1946 to March 2012), EMBASE (1980 to March 2012), Inspec (1969 to March 2012), International Pharmaceutical Abstracts (1970 to March 2012), PsycINFO (1806 to March 2012) all on the OvidSP platform, and CINHAL (1986 to March 2012) on EBSCOHOST platform. Secondary sources such as online database Pharmacy Abstracts, COCHRANE database, website of the Agency for Healthcare Quality (AHRQ), reference lists of included studies and Internet search engines were also searched. Keywords for the searches included “alerts, warning, decision support, pharmacist, pharmacy, expert system, pharmacy information systems, PMR systems and pharmacy order entry”. The search terms were initially used in MEDLINE and tailored to other databases. Citations were retrieved into Endnote X5. Details of the search strategy used for the MEDLINE search are provided in the Appendix section.

#### Study selection and data extraction

Two reviewers (OO and VG) independently screened the title and abstracts of articles to identify studies, which met our inclusion criteria. The full articles for the potentially relevant studies were retrieved and reviewed by three reviewers (OO, MB and AA) based on study type, study design, participants, settings, eligible outcomes and interventions. A hierarchical template was used to exclude non-relevant studies. No language restrictions were applied to the study selection process. One investigator (OO) designed and used a template to extract data from the articles included in the synthesis. Two reviewers (MB and AA) verified the extracted data. We resolved discrepancies by discussion until consensus was reached.

### Risk of bias in included studies

Two reviewers (OO and VG) independently assessed the quality of the included studies using the Cochrane Collaboration’s risk of bias tool [31]. The studies included in the synthesis were assessed individually for selection bias, allocation sequence concealment, blinding of participants and personnel, blinding of outcome assessment, incomplete outcome data, selective reporting and other bias.

## Results

**Figure 1:**
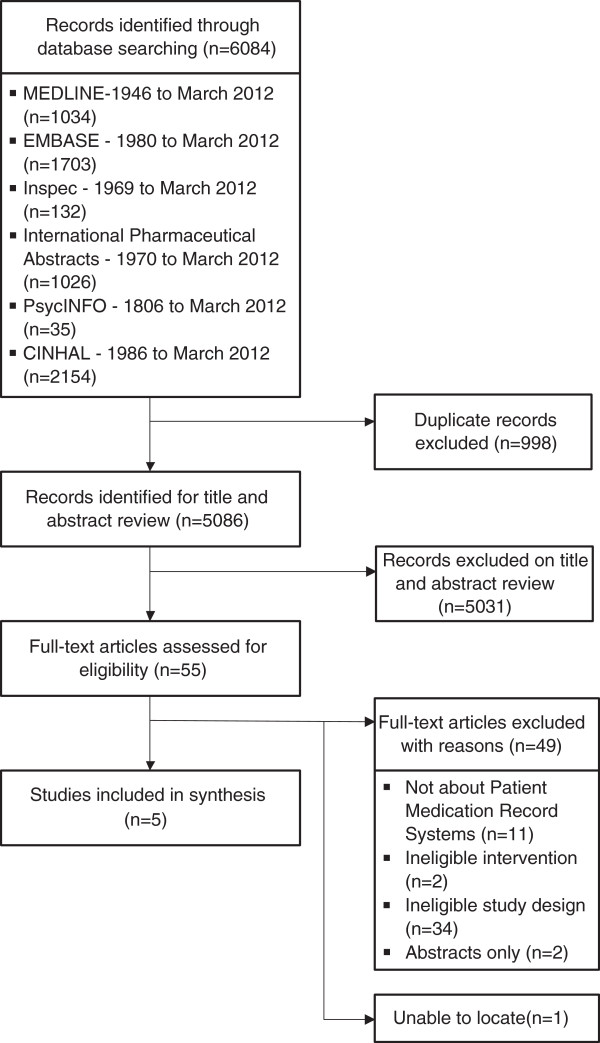
Flow diagram of included and excluded studies.

The literature search returned 6084 articles: MEDLINE (1034), EMBASE (1703), Inspec (132), International Pharmaceutical Abstracts (1026), PsychINFO (35) and CINAHL (2154). None of the secondary sources searched returned any relevant article. Two reviewers (OO and VG) reviewed the title and abstracts of the 5086, which remained after removing duplicate articles. A total of 5031 citations did not meet our inclusion criteria and were excluded. We were unable to locate one article [32]. The full articles of the remaining 54 citations were retrieved and based on the set criteria, 49 articles were excluded, leaving five eligible articles for the review. The excluded articles were not about PMR systems (11), contained ineligible interventions (2) or study designs (34), or were abstracts (2). Figure 1 shows the summary of this information. Table 1 is a summary of the characteristics of the included studies and the key findings.

**Table 1:** Characteristics of studies and alerts included in the systematic review

**Study**	**Study type**	**Intervention**	Setting*	**Country**	**Outcomes measured**	Alert functionality**	**Improved primary outcome (Yes/No)**	False positive alerts reported*** (Yes/No)	**Note**
Bhardwaja et al. 2010 [33]	RCT	Drug-lab alert (Renal)	HMO	US	Proportion of medication errors in drug selection or dosing of targeted drugs	Medication decision guide printed in lieu of the prescription label.	Yes	Yes	Pharmacists were trained to ensure effective communication of the reason for alerts and the rationale for drug changes to prescribers and patients. All activities were documented electronically.
Raebel et al. 2007 [34]	RCT	Drug-pregnancy alert	HMO	US	Primary - proportion of pregnant women dispensed a FDA category D or X medication. Secondary - total number of first dispensing of targeted medications	Prescription label not printed until pharmacist intervened.	Yes	Yes	False positive alerts led to early cancellation of study. Specific intervention guideline and patient counselling script were developed.
Raebel et al. 2007 [20]	RCT	Drug-age alert	HMO	US	Proportion of first dispensing of medications on the targeted medication list	Prescription label not printed until pharmacist intervened.	Yes	Yes	Intervention guideline and patient counselling script were developed. Pharmacists were required to complete an intervention note in the system before being able to print the prescription label. Notes were reviewed retrospectively.
Humphries et al. 2007 [21]	Before-After study (no control)	Drug interaction alert (Contraindicated)	HMO	US	Proportion of patients co-dispensed two critically interacting drugs	Prescription label not printed, pharmacist must consult with the prescriber.	Yes	…	Pharmacists could bypass the alert. They documented their activities electronically. Decision support guide was developed to aid them in interpreting and resolving critical alerts. Scripted conversations were used to explain to patients the reason for alerts and the rationale for medication changes.
Mansour et al. 2010 [14]	Before-After study (no control)	Drug-lab alert	Large teaching hospital (504-bed)	US	Proportion of hospitalised patients requiring treatment for hyperkalaemia	Pop-up alert about patient’s last reported potassium level	Yes	…	Pharmacists documented their response to the alert electronically. They could override the alert but were required to provide a reason for doing so. Number of alerts was retrieved through a report generated by the information technology pharmacist. Inconsistent response to the alert led to the development of the hyperkalaemia treatment guideline.

* HMO – Health Maintenance Organisation, FDA – Food and Drug Administration, RCT – Randomised Controlled Trial.** Alert generated when a trigger medication order was entered. *** Ellipses indicate false positive alerts were not reported.

### Description of included studies, baseline characteristics of patients and alerts

All the studies were from the US. Four of the five studies were conducted in an integrated care facility (Health Maintenance Organisation (HMO)) [20,21,33,34] and one in a hospital [14]. Three studies were randomised controlled trials (RCTs) [20,33,34], while two were before-after intervention design studies [14,21]. The publication dates for the studies ranged from 2007–2011. Four studies [20,21,33,34] compared baseline characteristics of patients but only one explicitly reported no significant differences in patients’ characteristics at baseline [33]. The five studies evaluated alerts to contraindicated prescribing in the following categories: drug-drug [21], drug-laboratory [14,33], drug-pregnancy [34] and drug-age [20].

Drug-drug interaction alert [21] generated when administration of a drug with another drug is likely to result in an increase or decrease in the action of either drug or result in an adverse effect that is not normally associated with either drug. Drug-lab alert [14,33] is generated when administration of a drug requires close monitoring of certain physiological parameters both before and after administration. A drug-pregnancy alert [34] is generated when administration of a drug is contraindicated in pregnancy while a drug-age alert [20] relates to a warning to the user of the system that certain medications might be contraindicated based on the age of the patient. All four alerts are similar to alerts normally found in computerised prescriber order entry (CPOE) systems but are notified to the pharmacist or user of ePMR system at the point of pharmacy order entry or dispensing.

### Risk of bias in included studies

The results of the risk of bias assessment of included studies are as shown in Table 2 (see Additional file 1 for derivations for these results). Of the five studies reported, three (RCTs) used computer generated sequence for randomisation and reported blinding of staff and participants to group assignment so selection and performance bias were judged as being a low risk. Allocation concealment was judged as unclear risk as it was not reported in any of the trials. The two before-after studies were judged to be at high risk of selection, sequence concealment and performance bias.

**Table 2:** Risk of bias summary

**Study**	**Allocation (selection bias)**	**Allocation sequence concealment**	**Blinding of participants and staff (performance bias)**	**Blinding of outcome assessment (detection bias)**	**Incomplete outcome data addressed**	**Free of selective reporting**	**Free of other bias**
Bhardwaja et al. 2010 [33]	Low	Unclear	Low	Low	Low	Low	Unclear
Raebel et al. 2007 [34]	Low	Unclear	Low	Unclear	Low	Low	Unclear
Raebel et al. 2007 [20]	Low	Unclear	Low	Unclear	Low	Low	Unclear
Humphries et al. 2007 [21]	High	High	High	Unclear	Low	Low	Unclear
Mansour et al. 2010 [14]	High	High	High	Unclear	Low	Low	Unclear

Assessment of the primary outcome measure was conducted in one of the studies and the study [33] was judged to be of low risk of detection bias. The risk of bias for the remaining four studies was judged as unclear. All the studies were judged as having a low risk of attrition and reporting bias. The three RCT studies reported false-positive alerts (alert wrongly indicating the presence of the attribute for which the alert should trigger) [20,33,34]. It was not clear if this could have introduced other biases into the studies so they were all judged to be of unclear risk of other bias. The two before-after studies were also judged as being of unclear risk of other bias because of the study design used.

### Analysis of intervention effect

The interventions had positive effects on outcomes in all the five studies.

#### Drug-drug interaction alert

In the study by Humphries, et al. [21], the primary outcome measure was the proportion of patients co-dispensed two critically interacting drugs. A 29% relative reduction in dispensing of critically interacting drugs was reported in the post-alert period, an average of 11.8 co-dispensing per 10,000 prescriptions over the entire post-alert period versus the pre-alert period (24.7 per 10,000 prescriptions). The overall rate of co-dispensing in the eight targeted drug pairs dropped sharply, from 21.3 to 14.7 per 10,000 prescriptions (p = 0.0125), representing a relative decrease in co-dispensing of 31% (95% CI −49.5 to −12.7) from the month before alert was implemented.

#### Drug-laboratory alert

The results of the two studies: a RCT [33] and a before-after study [14] featuring drug-laboratory alerts indicated that the intervention had a beneficial effect. The primary outcome in the RCT [33], was the proportion of medication errors defined as target drugs that should be avoided or were dosed inappropriately. In the usual care group, 1853 patient-drug combinations were dosed inappropriately (49%, 95% confidence interval (CI) 47–50%) out of 3796. In the intervention group, 1195 patient-drug combinations were dosed inappropriately (33%, 95% CI 31–34%) out of 3639 which was significantly lower than the usual care group (p<0.001). With regard to target drugs that required a dosage adjustment, 1291 of the 3231 patient-drug combinations in the usual care group were dosed inappropriately (40%, 95% CI 38–42%). In the intervention group, 893 (27%, 95% CI 25–28%) out of 3335 patient-drug combinations were dosed inappropriately, which was considerably lower than the usual care group (p<0.001).

In the before-after study [14], the alert warned pharmacists about potassium level above the set value and was tracked 63 times over the 3-month alert implementation period with the largest number of cases occurring during order entry for a potassium supplement (20 cases) and an ACE inhibitor (34 cases). Prior to implementation of alert, 48 hospitalized patients were treated for hyperkalaemia but only 14 cases of hyperkalaemia required treatment after the alert was implemented, a statistically significant decrease in ADEs related to hyperkalaemia (p<0.001).

#### Drug-pregnancy alert

The primary outcome of the study by Raebel, et al. [34] was the proportion of pregnant women dispensed US FDA category D (evidence of fetal risk exists, but therapeutic benefits can outweigh the risk) or X (evidence from human or animal studies suggests that risk to the fetus outweighs therapeutic benefit) medications. A total of 177 (2.9%) women in the intervention group were dispensed at least one medication from category D or X compared with 276 (5.5%) women in the usual care group (p<0.001).

#### Drug-age alert

The intervention by Raebel, et al. [20] led to a decrease in the proportion of patients aged 65 and over who were newly prescribed potentially inappropriate medications. In the analysis of dispensing of medications to patients aged 65 and over, 543 of 29,840 (1.8%) patients randomised to the intervention group were newly dispensed at least one potentially inappropriate medication compared to 644 of 29,840 (2.2%) in the usual care group (p=0.002, 16% relative risk reduction).

### False alerts and alert management

The three RCT studies reported problems of false-positive alerts [20,33,34]. In the study by Bhardwaja et al. [16], false-positive alerts determined as the proportion of alerts that had a creatinine clearance level > 51 ml/min on medical record review, reduced from 32% to 0.5% one year after the alert was changed to drug-specific creatinine clearance cut-off. The study by Raebel, et al. [34] was terminated early because of false-positive alerts due to misidentification of medications as contraindicated in pregnancy and incorrect alert that patients were pregnant. In another study [20], alerts for excluded indications were considered to be false-positives but the authors did not indicate the extent of the problem.

Mansour et al. [14] noted a lack of consistency in the way pharmacists managed alerts during the first few months following alert implementation. The management team then developed the hyperkalaemia treatment guidelines to assist pharmacists when deciding whether to dispense the prescribed medication and what suggestions to give to the prescribing physician when calling regarding the hyperkalaemia alert.

In four of the studies, the medication alerts functioned by preventing the prescription label from being printed until the pharmacist had actively intervened to determine whether the prescription should be dispensed or whether to contact the prescriber [20,21,33,34]. In one study, a decision support guide was printed for the pharmacist in lieu of the prescription label [33]. In another, a pop-up screen appeared on screen, warning pharmacists about patient’s last reported potassium level, including a reminder regarding where to document the response to the alert. Pharmacists could override alerts as long as the reason for the override was explained and the action documented [14].

## Discussion

We identified five studies and each of these showed that using alerts in ePMR systems resulted in reductions in dispensing of critically interacting, contraindicated drugs based on age or pregnancy, adverse drug events related to hyperkalaemia and medication errors. Thus, ePMR systems have the potential to improve patient safety by processing personal and clinical information along with medication records to provide patient specific advice. This can help to reduce the risks that may arise from taking prescribed or non-prescribed medicines. Nevertheless, while the interventions had a positive effect on primary outcomes in the studies we identified, there were instances of false alerts and discrepancies in alert management.

### Comparisons with other studies

Results from this review support some of the findings from other studies that have looked at the performance of safety features in ePMR systems. It has been suggested that only known hazards and not theoretical possibilities should be listed as contraindicated drug-drug combinations and that alerts are taken seriously if users are alerted to situations that require action in order to improve patient safety [35]. This is a plausible explanation for why the evaluated studies focused on specific instances and group of drugs.

### False alert, intervention guideline and alert management

Problems of false-positive alerts appear to be prevalent [20,33,34]. This may result from programming deficiencies [25], lack of required information, misclassification of alerts [34], among others leading to reduction in specificity and sensitivity of alerts. False alerts, and alerts that are not clinically significant, may result in pharmacists ignoring clinically important alerts [36-38]. It has been reported that pharmacists often override alerts generated about drug-drug interactions which they receive [39] and information and advice from the alerts are often ignored [40,41]. One of the studies was terminated early because of false-positive alerts [34].

Active collaboration between pharmacists, physicians, and other relevant stakeholders is necessary when developing target medication lists and indications for which an intervention should occur. Collaborative development of intervention guidelines and implementation of the intervention are also some of the ways in which stakeholders can work together to make alerts more relevant, useful and effective in practice thereby guaranteeing the success of any intervention programme.

Linking patient medication records with other clinical data when possible expands the scope of checks for clinically hazardous situations at the point of pharmacy order entry [20,34]. However, inadequate knowledge of the safety features available or necessary in ePMR systems coupled with lack of training on how to deal with some alerts can result in sub-optimal use of the safety features in ePMR systems. Development of treatment and alert-response guidelines, decision support guides, and training offer an opportunity to ensure consistency in the way pharmacists intervene or manage alerts.

Evaluating cases where pharmacists did not adhere to recommendations in alerts and the reasons for non-adherence would be useful in fine-tuning alerts during future developments to enhance the effectiveness of safety alerts and prevent over-alerting and alert fatigue. Results from observational studies have shown that pharmacists override or ignore alerts. In one observational study, pharmacists expressed lack of trust in the clinical significance and accuracy of the alert information consequently leading to alerts being overridden [40]. Indermitte et al. [36], found that alerts for potential drug interactions were graded as severe, moderate and minor. Pharmacists could select the levels of alerts to flag in the system during order entry leading to variation in the systems and overriding of alerts that were considered insignificant. User friendliness of the systems can be increased by customisation but may also lead to variation in the system [13]. Lack of an agreed standard and consensus about severity levels for drug interactions is a major reason for the variation, which exists between ePMR systems, as vendors have no choice than to rely on their preferred or own classification system [15].

The evaluated safety features were created based on the needs of the respective institutions and a desire to prevent adverse events in various clinical situations. This suggests that safety features embedded in ePMR systems in different domains may not always be the same. This has implications for commercial ePMR systems, as they may not always meet the needs of users in every domain without customisation. Although customisation of alerts is not often possible in primary care pharmacy, ePMR users should be alerted about ‘never events’ involving medications and have the option to choose what they would like to be alerted about to prevent over-alerting and automatic behaviour towards alerts.

### Strengths and limitations

One of the strengths of this systematic review is its rigorousness. It is also the first one in the field, to our knowledge, that has examined the effectiveness of safety features and alerts in ePMR systems at the point of pharmacy order entry in primary and secondary care.

There are some limitations to this review. Alerts on non-pharmacy systems are beyond the scope of this review. Although pharmacists managed the alerts in the studies evaluated in this review, this may not be a true reflection of the practice situation in some countries and practice domains. It is often the case that the person processing a medication order is not the pharmacist. Some pharmacists allow their support staff to override alerts up to a certain degree of severity without an initial discussion with the pharmacist. In such circumstances, it is important to keep the pharmacist informed about important alerts if someone else handles the prescription. There have been concerns that important interactions could be missed if dispensing staff do not alert pharmacists appropriately [39].

### Implications for policy and practice

The point of pharmacy order entry is regarded as a safety net in preventing medication error and adverse drug events (ADEs) once a prescription has been issued [42,43]. This systematic review provides evidence that alerts are useful for picking up medication errors and potential clinical hazards at the point of pharmacy order entry. From a policy perspective, it is important to consider strengthening regulations around the provision of safety features in ePMR systems. It is also important to ensure that such systems are designed in a way that maximises the likelihood of pharmacists taking appropriate actions. Increasing the specificity of alerts by taking into account individual patient context will help reduce the number of false alerts leading to alert bypass. From a practice perspective, it is important that pharmacists recognise the value of ePMR systems in preventing the dispensing of hazardous prescriptions, and therefore pay attention to electronic alerts even if over-alerting remains a problem for some time to come.

### Further research

Although this review provided some insight into the effectiveness of safety features and alerts in ePMR systems, it has also highlighted gaps in this subject area. The review did not identify any study that has evaluated the effectiveness of other safety features that may be found in ePMR systems such as safety features for screening for therapeutic duplication, allergies, adherence issues, excessive and sub-optimal doses, inappropriate prescribing and drug-drug interactions (non-contraindicated) at the point of pharmacy order entry. All the studies were from the US and none from community pharmacy. We also do not know the influence of various design features on user-acceptance of alerts. These gaps in the literature need addressing.

The impact of and handling of alerts by migrant pharmacists and cross-sector pharmacists who often have to use ePMR systems that they may not be familiar with is yet to be explored. Generating evidence in this area will help to promote an understanding of the challenges faced by migrant pharmacists to prevent error promoting situations that may arise when using such systems.

More research needs to be done to investigate the impact of alert customisation and standards for ePMR systems and alerts on patient safety. Some alerts will not flag when prescriptions are dispensed from pharmacies that a patient does not use regularly, if the relevant ePMR system does not have the historical medication record of that patient. The potential patient safety benefits from making information about medications prescribed to patients and other details such as patients’ allergy status, available to the dispensing pharmacy at the point of order entry should be explored.

## Conclusions

This systematic review shows that ePMR systems in conjunction with the embedded safety features are effective in picking up and reducing potential problems and clinical hazards at the point of pharmacy order entry. There are, however, problems of false alerts and inconsistencies in alert management. Due to low number of relevant studies, there was insufficient evidence to enable adequate comparison of the safety features evaluated across practice domains or studies. More studies are needed to assess the effectiveness of other safety features that are present in ePMR systems such as dosing guidance based on age, therapeutic duplication, and drug-allergy screening. Design features associated with safety alert acceptance and effectiveness should be evaluated. Finding appropriate solutions to these problems is a challenge but it is of paramount importance to strengthen the safety net and continue to prevent harm to patients.

## Appendix

### Search protocol used for identifying studies in Medline

1. (e adj2 prescrip$).mp. [mp=title, abstract, original title, name of substance word, subject heading word, unique identifier]

2. (elec$ adj prescrip$).mp. [mp=title, abstract, original title, name of substance word, subject heading word, unique identifier]

3. exp Drug Labeling/

4. Drug Utili?ation Review.mp. [mp=title, abstract, original title, name of substance word, subject heading word, unique identifier]

5. Pharmacy Information System$.mp.

6. Patient Medication Record$.mp. [mp=title, abstract, original title, name of substance word, subject heading word, unique identifier]

7. (Pharmacy or Pharmacies or Pharmacist$).mp. [mp=title, abstract, original title, name of substance word, subject heading word, unique identifier]

8. 1 or 2 or 3 or 4 or 5 or 6 or 7

9. (Safety adj2 (Alert$ or Feature$ or Alarm$ or Warning$ or Prompt$)).mp. [mp=title, abstract, original title, name of substance word, subject heading word, unique identifier]

10. (Over?rid$ adj25 Alert$).mp. [mp=title, abstract, original title, name of substance word, subject heading word, unique identifier]

11. Reminder System$/

12. alert$.mp.

13. Decision Support$.mp.

14. exp Decision Making, Computer-Assisted/

15. exp Drug Dosage Calculations/

16. 9 or 10 or 11 or 12 or 13 or 14 or 15

17. 8 and 16

## Competing interests

The authors declare that they have no competing interests.

## Authors’ contributions

OO, AA MB designed the review. OO developed the search strategy with support from AA and MB. OO ran the searches. OO and VG reviewed the titles and abstracts. OO, AA and MB reviewed the full papers to determine which to include in the review. OO abstracted the data from the included studies and this was checked by MB. OO and VG assessed the risk of bias for the included studies. All authors were involved in the interpretation of the findings. OO wrote the first draft of the paper and AA, MB and VG critically reviewed this. All authors read and approved the final manuscript.

## Pre-publication history

The pre-publication history for this paper can be accessed here:

http://www.biomedcentral.com/1472-6947/13/69/prepub

## Supplementary Material

Additional file 1: Table S1 Risk of bias assessment. Click here for file
