# Supplementary material for: The evidence for the effectiveness of safety alerts in electronic patient medication record systems at the point of pharmacy order entry: a systematic review
Source: BMC Med Inform Decis Mak. 2013 Jul 1;13:69. doi: 10.1186/1472-6947-13-69 (PMC3702525; doi:10.1186/1472-6947-13-69)
Supplement: Additional file 1: Table S1 — Risk of bias assessment. [file 1472-6947-13-69-S1.docx]

**Risk of bias assessment**

| **Bhardwaja *et al.* 2010 [33]** |  |  |
| --- | --- | --- |
| **Bias** | **Authors’ judgement** | **Support for judgement** |
| Allocation (selection bias) | Low risk | Quote: “…the entire membership of KPCO was randomly assigned to either the usual care group or the intervention group…by using the uniform distribution function in SAS…” |
| Allocation sequence concealment | Unclear risk | Comment: Not reported |
| Blinding of participants and personnel (performance bias) | Low risk | Quote: “The study subjects, study investigators and study personnel were blinded to group assignments” |
| Blinding of outcome assessment (detection bias) | Low risk | Quote: “…a research pharmacist blinded to group assignments performed a medical record review of all target drug dispensing to patients in the intervention group…” |
| Incomplete outcome data (attrition bias) | Low risk | Comment: None were identified |
| Selective reporting (reporting bias) | Low risk | Comment: None were identified |
| Other bias | Unclear risk | Comment: Randomisation was unbalanced.  (2) False positive alert types  Quote: “At the beginning of the study, the number of false-positive alerts (determined as the proportion of alerts that had Clcr > 51 ml/min on medical record review) was almost 32%. One year after changing the alert to the drug-specific Clcr cut-off, the false-positive rate was reduced to 0.5%.” |

| **Humphries *et al.* 2007 [21]** |  |  |
| --- | --- | --- |
| **Bias** | **Authors’ judgement** | **Support for judgement** |
| Allocation (selection bias) | High risk | Comment: Not randomised |
| Allocation sequence concealment | High risk | Comment: Not reported |
| Blinding of participants and personnel (performance bias) | High risk | Comment: Blinding not described |
| Blinding of outcome assessment (detection bias) | Unclear risk | Comment: Blinding of outcome assessors was not described |
| Incomplete outcome data (attrition bias) | Low risk | Comment: None were identified |
| Selective reporting (reporting bias) | Low risk | Comment: None were identified |
| Other bias | Unclear risk | Comment: Bias related to before-after study design |

| **Mansour *et al.* 2010 [14]** |  |  |
| --- | --- | --- |
| **Bias** | **Authors’ judgement** | **Support for judgement** |
| Allocation (selection bias) | High risk | Comment: Not randomised |
| Allocation sequence concealment | High risk | Comment: Not randomised |
| Blinding of participants and personnel (performance bias) | High risk | Comment: Before-after study, blinding not described |
| Blinding of outcome assessment (detection bias) | Unclear risk | Comment: Blinding of outcome assessors was not described |
| Incomplete outcome data (attrition bias) | Low risk | Comment: None were identified |
| Selective reporting (reporting bias) | Low risk | Comment: None were identified |
| Other bias | Unclear risk | Comment: Bias related to before-after study design |

| **Raebel *et al.* 2007 [34]** |  |  |
| --- | --- | --- |
| **Bias** | **Authors’ judgement** | **Support for judgement** |
| Allocation (selection bias) | Low risk | Quote: “…approximately 340,000 individuals were randomized (using the uniform distribution function in SAS…”  Comment: Randomisation was unbalanced. It is unlikely to have had any negative impact on group assignment |
| Allocation sequence concealment | Unclear risk | Comment: Not reported |
| Blinding of participants and personnel (performance bias) | Low risk | Quote: “Physicians, patients and pharmacists were blinded to study group assignment” |
| Blinding of outcome assessment (detection bias) | Unclear risk | Comment: Blinding of outcome assessors was not described |
| Incomplete outcome data (attrition bias) | Low risk | Comment: None were identified |
| Selective reporting (reporting bias) | Low risk | Comment: None were identified |
| Other bias | Unclear risk | Comment: (1) Randomisation was unbalanced. (2) Study was stopped early due to false-positive alert types but it is unclear if this could have introduced other biases into the study. |

| **Raebel *et al.* 2007 [20]** |  |  |
| --- | --- | --- |
| **Bias** | **Authors’ judgement** | **Support for judgement** |
| Allocation (selection bias) | Low risk | Quote: “…the entire membership of KPCO was randomized to an intervention or usual care group using the uniform distribution function in SAS” |
| Allocation sequence concealment | Unclear risk | Comment: Not reported |
| Blinding of participants and personnel (performance bias) | Low risk | Quote: “Physicians, patients and pharmacists were blinded to study group assignment” |
| Blinding of outcome assessment (detection bias) | Unclear risk | Comment: Blinding of outcome assessors was not described |
| Incomplete outcome data (attrition bias) | Low risk | Comment: None were identified |
| Selective reporting (reporting bias) | Low risk | Comment: None were identified |
| Other bias | Unclear risk | Comment: False positive alerts  Quote: “In the context of this study, alerts for excluded indications could be considered false-positives, because the pharmacist reviewed the prescriptions to determine that the alerts were for excluded indications and documented that, but the pharmacist did not intervene” |
